# Quantifying patch‐specific seed dispersal and local population dynamics to estimate population spread of an endangered plant species

**DOI:** 10.1002/ece3.8116

**Published:** 2021-09-14

**Authors:** Jinlei Zhu, Karolína Hrušková, Hana Pánková, Zuzana Münzbergová

**Affiliations:** ^1^ Institute of Botany Czech Academy of Sciences Průhonice Czech Republic; ^2^ Institute of Landscape and Plant Ecology University of Hohenheim Stuttgart Germany; ^3^ Department of Botany Faculty of Science Charles University Prague Czech Republic

**Keywords:** Habitat fragmentation, in situ conservation, population dynamics, stage‐structured matrix population model, WALD mechanistic model, wave speed model

## Abstract

**Aim:**

Habitat loss and fragmentation impose high extinction risk upon endangered plant species globally. For many endangered plant species, as the remnant habitats become smaller and more fragmented, it is vital to estimate the population spread rate of small patches in order to effectively manage and preserve them for potential future range expansion. However, population spread rate has rarely been quantified at the patch level to inform conservation strategies and management decisions. To close this gap, we quantify the patch‐specific seed dispersal and local population dynamics of *Minuartia smejkalii*, which is a critically endangered plant species endemic in the Czech Republic and is of urgent conservation concern.

**Location:**

Želivka and Hrnčíře, Czechia.

**Methods:**

We conducted demographic analyses using population projection matrices with long‐term demographic data and used an analytic mechanistic dispersal model to simulate seed dispersal. We then used information on local population dynamics and seed dispersal to estimate the population spread rate and compared the relative contributions of seed dispersal and population growth rate to the population spread rate.

**Results:**

We found that although both seed dispersal and population growth rate in *M. smejkalii* were critically limited, the population spread rate depended more strongly on the maximal dispersal distance than on the population growth rate.

**Main conclusions:**

We recommend conservationists to largely increase the dispersal distance of *M. smejkalii*. Generally, efforts made to increase seed dispersal ability could largely raise efficiency and effectiveness of conservation actions for critically endangered plant species.

## Graphical abstract

To inform conservation strategies and management decisions of the critically endangered *Minuartia smejkalii*, we quantify its patch‐specific population spread rate by determining seed dispersal and local population dynamics in each remnant patch. We found that although both seed dispersal and population growth rate in *M. smejkalii* were critically limited, the population spread rate depended more strongly on the maximal dispersal distance than on the population growth rate. We recommend conservationists to largely increase the dispersal distance of *M. smejkalii*, and we believe that efforts made to increase seed dispersal ability could largely raise efficiency and effectiveness of conservation actions for critically endangered plant species in general.

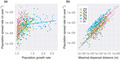

## 1 INTRODUCTION

There is an urgent need to conserve numerous endangered plant species globally (Ceballos et al., 2015; Halley et al., 2016; Newbold et al., 2015; Urban, 2015). Many of these species are restricted by habitat loss and fragmentation (Heinken & Weber, 2013; Yesuf et al., 2021) leading to their possible dispersal limitation (Bonte et al., 2012; Emsens et al., 2018; Sun et al., 2020). An important prerequisite for effective conservation of these species is to understand population growth and spread rates of these species and their determinants. Population growth and spread rates strongly depend on local population dynamics and dispersal (Fisher, 1937; Kot et al., 1996; Neubert & Caswell, 2000; Okubo & Levin, 1989; Schreiber & Beckman, 2020; Skellam, 1951). A range of recent studies dealt with local population dynamics of many endangered plant species (e.g., Alexander et al., 2018; Andrieu et al., 2017; Bucharová et al., 2012; Dostálek & Münzbergová, 2013; Heinken‐Šmídová & Münzbergová, 2012; Marrero et al., 2019). However, much less is known about dispersal abilities of these species. The knowledge on these two processes provides important bases of models describing species dynamics at the landscape level (e.g., Bullock et al., 2020; Mildén et al., 2006; Münzbergová et al., 2005; Quintana‐Ascencio et al., 2018). However, only a few studies attempted to combine the information on population dynamics and dispersal to assess the possible spread rates of the species (Block & Levine, 2021; Bullock et al., 2012, 2020; Hemrová et al., 2017; Jongejans et al., 2008), limiting our ability to estimate species dynamics at the regional scale.

The knowledge on population spread rate is crucial for understanding species ability to expand in remnant patches for two reasons. First, comparing the patch‐specific population spread rate will help conservationists to identify those patches where range expansion needs to be promoted. Second, disentangling the effects of local population dynamics and seed dispersal on the population spread rate will clarify the critical factors and life‐history stages most threatening range expansion. For example, if the low population spread rate is mainly due to limited dispersal ability, efforts should be made to increase effective seed dispersal. While such information may be useful in many conservation actions, we are not aware of any study which would attempt to perform such a comparison.

*Minuartia smejkalii* is a critically endangered plant species endemic in the Czech Republic. The species is adapted to stressful habitats with serpentine soils (Lozada‐Gobilard et al., 2020; Stojanova et al., 2020, 2021), which have low essential nutrients and organic content but high concentrations of heavy metals (Chiarucci & Baker, 2007). Widely existing in steep and rocky areas that are prone to erosion, serpentine soils are often rather shallow and nutrient‐poor. Together with sparse vegetation, high soil temperatures, and drought, serpentine soils can create strong selective pressures on plants, leading to small populations, low population growth rates, and high endemism (Brady et al., 2005). The species is a shade‐intolerant herbaceous perennial and a weak competitor. Although surviving in highly fragmented habitats, the species has low genetic differentiation (Stojanova et al., 2020) and does not show early inbreeding depression (Stojanova et al., 2021). This may suggest that the species might have high levels of gene flow between populations. Alternatively, it may reflect conditions in the past, when the populations used to be more continuous, rather than at present, due to species longevity. Indeed, such stronger effects of past rather than current conditions on genetic diversity of species have been shown in several previous studies (e.g., Aavik et al., 2019; Münzbergová, 2013; Plue et al., 2017). The latter explanation is also more likely given that the plants grow mostly shorter than 20 cm and produce tiny seeds that lack special appendages that assist seed dispersal by wind or animals, leading to short primary seed dispersal distances (Lozada‐Gobilard et al., 2020). These traits suggest that the species might face severe dispersal limitation. The nutrient‐poor habitats and life‐history traits of *M. smejkalii* make the species a representative endangered plant species in conservation biology. Therefore, studying population spread of *M. smejkalii* may help to better understand how endangered plant species with limited seed dispersal ability spread in harsh environment in general.

The motivation behind this study is to understand whether *M. smejkalii* can successfully colonize new suitable sites in nature. The study has two aims: first, to estimate the population spread rate of *M. smejkalii*; and second, to determine the most critical factors limiting the population spread rate. Specifically, we address four questions: (a) What is the population spread rate in *M. smejkalii* in different patches? b) How far can seeds of *M. smejkalii* be dispersed by wind in different patches? (c) How does the population growth rate of *M. smejkalii* vary in different patches? and (d) how do dispersal distance and population growth rate affect population spread rate in *M. smejkalii*?

## 2 METHODS

### 2.1 Study species

*Minuartia smejkalii* is only occurring in the Czech Republic in Želivka and Hrnčíře regions with a total area of 500 km^2^ (Bilz et al., 2011; Lozada‐Gobilard et al., 2020; Stojanova et al., 2020). The species is threatened by habitat loss and fragmentation caused by mining activities, construction of motorway and water reservoir, and agricultural intensification since the 1960s (Stojanova et al., 2020). *Minuartia smejkalii* occurs in Hrnčíře in one patch and in Želivka in six patches, and 1,442 individuals in total were found in the wild in 2020. The species is classified as “critically endangered” in the Czech Republic and listed in Appendix S1 of the Convention on the Conservation of European Wildlife and Natural Habitats and the IUCN international Red List (Bilz et al., 2011), and belongs to the species of priority European interest according to Habitats Directive 92/43/EEC, Annex II. It has high genetic diversity due to outcrossing mating systems, but shows low genetic differentiation despite high fragmentation (Stojanova et al., 2020). In this study, we selected five out of six existing patches, due to small size of the remaining patch, four in the Želivka region Z2, Z4, Z5, and Z6, and the only one in the Hrnčíře region (H), to estimate population spread rate of *M. smejkalii*.

### 2.2 Dispersal environment

The most important biological and physical dispersal environments determining wind dispersal are surrounding vegetation height and horizontal wind velocity, respectively. Vegetation surrounding *M. smejkalii* is sparse in nature, and vegetation height was generally lower than 5 cm. Horizontal mean wind velocity at the reference height of 10 m was acquired from one weather station in each region, Hulice for the Želivka region and Košetice for the Hrnčíře region (www.chmi.cz), and Weibull distributions were fitted to the wind measurements (Seguro & Lambert, 2000). Other parameters aerodynamic roughness length and friction velocity, which were required to determine the wind profile (vertical distribution of horizontal mean wind speed), were calculated following Skarpaas and Shea (2007). With these parameters, we fitted logarithmic wind velocity profiles to calculate horizontal wind velocity at any height of interest (Bullock et al., 2012; Monteith & Unsworth, 2013).

### 2.3 Dispersal traits

We determined two dispersal traits, seed release height and terminal velocity (the constant speed of a falling seed in still air (Tackenberg, 2003; Zhu et al., 2016). Seed release height was recorded as the height of seed heads above soil surface. For each population, we randomly selected 10–23 individual plants and measured 6–15 seed heads from each individual, resulting in 130–165 records per population, and in total measured 760 values from 70 individuals (Appendix Table S1). Variation in the number of measurements was due to availability of plant individuals and seed heads. We measured terminal velocity using ripe air‐dried seeds (seed mass: 0.051 ± 0.004 mg (mean ± *SD*)) from 5 to 12 individuals per population with a self‐built apparatus at the University of Hohenheim, Germany. The apparatus consists of an automatic seed release device, a high‐speed camera (acA1920‐155 um, BASLER) at a fixed speed of 130 frames per second, which shoots videos of a falling seed from two perspectives simultaneously. From the videos, we extracted the true 3D coordinates of the falling seed in space over time with ImageJ (Schneider et al., 2012), fitted a mechanistic acceleration model describing the whole falling process, and then calculated seed terminal velocity based on the laws of physics. We measured 33–70 values of seed terminal velocity for each patch, and in total measured 274 values from 42 individuals (Appendix Table S1). Variation in the number of measurements was due to seed availability. We fitted log‐normal distributions to the patch‐specific measurements of seed release height and seed terminal velocity and used these distributions in the simulations of seed dispersal (see below).

### 2.4 Data analysis

*Dispersal simulation*: We used the mechanistic Wald analytical long‐distance dispersal (WALD) model (Katul et al., 2005; Skarpaas & Shea, 2007) to simulate seed dispersal (Appendix S2). The model took patch‐specific parameters of biological and physical dispersal environments and dispersal traits as input (Appendix Table S2), and calculated seed dispersal distance and the probability of long‐distance dispersal (LDD) as output. In the simulations, neighboring vegetation was assumed to be homogeneous and vegetation height was randomly drawn from a log‐normal distribution with a mean of 3 cm and standard deviation of 0.002 cm. This simplification allowed to compare the simulations among patches. We chose a threshold of LDD as 0.5 m, in line with a recent study showing that 99% of seeds of *M. smejkalii* are dispersed shorter than 0.03 m and the maximal distance was less than 0.25 m (Lozada‐Gobilard et al., 2020). We simulated dispersal events of one million seeds for each patch with 10 replicates.

*Demographic analysis*: To gain better insights into the processes that drive the population dynamics of *M. smejkalii*, and the specific life‐history stages that played most important roles in population growth rates, we constructed 15 (5 patches × 3 years) stage‐structured population projection matrices (PPMs) from demographic data that were collected in the patches between 2016 and 2018 (three transitions), considering three different life‐history stages: seedlings, juveniles, and large adult plants (Caswell, 2000) (Appendix S1). Based on these 15 PPMs, we calculated three types of matrices for demographic analyses and estimation of population spread rates at different levels: first, an overall mean PPM of these 15 PPMs across all patches; second, a mean PPM of the three PPMs between 2016 and 2018 for each patch; and third, to compare the relative contributions of population growth rate and dispersal distance to the population spread rate, for each patch we “bootstrapped” 100 matrices from the three PPMs of that patch between 2016 and 2018. We mixed the stage‐specific values in the three PPMs, creating 19,683 (=3^9^) possible PPMs, and randomly sampled 100 PPMs whose eigenvalues were greater than one, ensuring that the corresponding population spread rates were larger than zero. We then used those 500 PPMs (100 PPMs/patch × 5 patches) to compute the population growth and spread rates. We computed the population growth rate, quasi‐extinction probability, and elasticity for each life‐history stage, using the R package popbio (Stubben & Milligan, 2007).

*Modeling of population spread*: We estimated population spread rate following Bullock et al. (2012) and Hemrová et al. (2017). We combined the analytical wavespeed model of Neubert and Caswell (2000) with a demographic matrix model with integrodifference equations describing dispersal (Gilbert et al., 2014; Appendix S3).

*Regression analysis*: To compare the relative contributions of population growth rate and the maximal dispersal distance to the population spread rate, we used linear models between the population spread rate and population growth rate and the maximal dispersal distance, respectively, and compared the coefficient of determination of the models. We scaled population growth rate and the maximal dispersal distance in the models, by using the function scale in R, to ensure that the variables with different units were comparable.

Data on seed dispersal and population spread are available on the Dryad Digital Repository (Zhu, 2021).

## 3 RESULTS

### 3.1 Population spread rate of *M. smejkalii*

**FIGURE 1:**
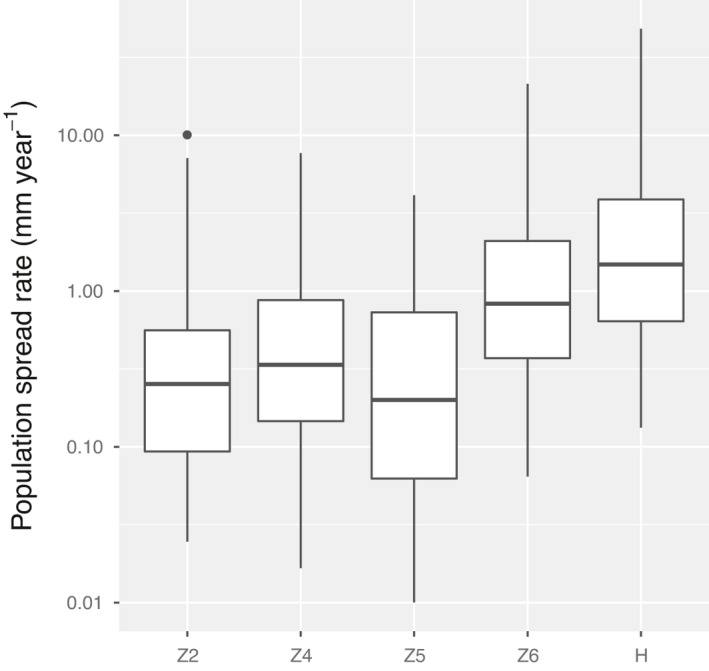
Population spread rate of *Minuartia smejkalii* in different remnant patches. Center lines show the medians; box limits indicate the 25th and 75th percentiles; whiskers extend 1.5 times the interquartile range from the 25th and 75th percentiles; and outliers are represented by dots. *n* = 100 sample points for each patch. Note that y‐axis was plotted on a logarithmic scale

There was a large variation in population spread rate among patches. Across all patches, median population spread rate was 1.2 mm/year, and the maximum population spread rate was 23.4 mm/year (Table 1). In the patch Z2, the population spread rate was zero, because population growth rate was less than one. In patches Z4, Z5, Z6, and H, where population growth rate was greater than one, the median population spread rate was below 2 mm/year and the maximum population spread rate was below 52 mm/year (Figure 1; Table 1).

**TABLE 1:** Demographic analysis and population spread rate of *Minuartia smejkalii*

Patch	population growth rate	Quasi‐extinction probability		Elasticity of survival			Population spread rate (mm/year)	
		in 20 years	in 50 years	S1	S2	S3	median	maximum
Z2	0.88	0.22 ± 0.01	1	0.10	0.29	0.52	0	0
Z4	1.05	0.001 ± 0.001	0.017 ± 0.004	0.15	0.37	0.33	0.4	5.3
Z5	1.04	0.21 ± 0.01	0.43 ± 0.03	0.11	0.40	0.39	0.2	2.1
Z6	1.66	0	0	0.30	0.14	0.19	1.3	25.7
H	1.17	0	0	0.17	0.22	0.43	1.8	51.5
Overall	1.27	0	0	0.23	0.26	0.27	1.2	23.4

Note

S1: seedling; S2: juvenile; S3: large adult plants. Elasticity of survival was sum of the second and third rows of the elasticity matrices of population growth rate, corresponding to S2 and S3, respectively (see the elasticity matrices of population growth rate in Appendix S4).

### 3.2 Seed dispersal distance and probability of LDD of *M. smejkalii*

**FIGURE 2:**
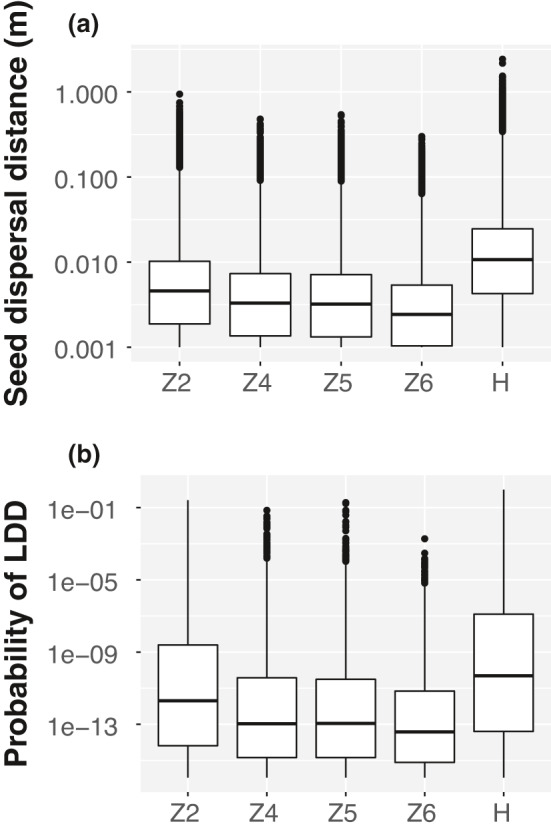
(a) Predicted seed dispersal distance and (b) probability of long‐distance seed dispersal (LDD) of *Minuartia smejkalii* in different remnant patches. Center lines show the medians; box limits indicate the 25th and 75th percentiles; whiskers extend 1.5 times the interquartile range from the 25th and 75th percentiles; and outliers are represented by dots. *n* = 10^7^ sample points for each patch. Note that y‐axis was plotted on a logarithmic scale

There was a large variation in seed dispersal distance of *M. smejkalii* among patches. The median seed dispersal distance was shorter than two centimeters (Figure 2a; Appendix Table S3); the maximal seed dispersal distance was shorter than one meter in the patches Z2, Z4, Z5, and Z6, and 2.5 m in the patch H (Appendix Table S3). The median probability of LDD was zero in all patches (Appendix Table S4), indicating that majority of seeds were only dispersed within the range of 0.5 m; the maximal probability of LDD was 26% in Želivka region (patch Z6) and 99% in the Hrnčíře (patch H) (Figure 2b), suggesting that certain seeds should be dispersed further than 0.5 m in Hrnčíře (Appendix Table S4).

### 3.3 Demographic analyses of *M. smejkalii*

Across all patches, the population growth rate was 1.27, suggesting that *M. smejkalii* can expand its population size. The quasi‐extinction probability was zero in 50 years, indicating population persistence. The largest elasticity was for survival of large adults (Table 1). In patches Z6 (population growth rate =1.66) and H (population growth rate =1.17), the quasi‐extinction probability was zero in 50 years, indicating population persistence. However, in patches Z4 (population growth rate =1.05) and Z5 (population growth rate =1.04), the species faces certain extinction risk. In contrast, population growth rate was 0.88 in the patch Z2, suggesting that the population was in decline. The quasi‐extinction probability in the patch Z2 was predicted to drastically increase in 15 years, and the population was projected to go extinct in 40 years. The largest elasticity was for survival of large adults in patches Z2 and H, for juveniles in patches Z4 and Z5, and for seedlings in the patch Z6 (Table 1).

### 3.4 *Effects of dispersal* versus *local population dynamics on population spread of M. smejkalii*

**FIGURE 3:**
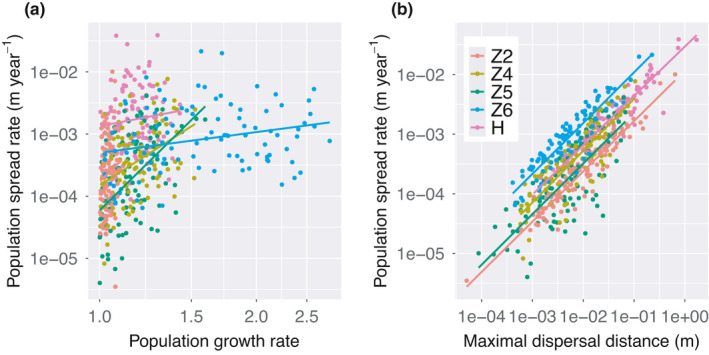
Relationships between the population spread rate and (a) the population growth rate and (b) the maximal seed dispersal distance of *Minuartia smejkalii* in different remnant patches. Note that both x‐axis and y‐axis were plotted on a logarithmic scale

The population spread rate depended more strongly on the maximal dispersal distance (*R* ^2^ ranging from 0.377 to 0.886) than on the population growth rate (*R* ^2^ ranging from 0.016 to 0.213) (Figure 3; Appendix Table S5). Across all patches, there was a significant positive correlation between the population spread rate and the maximal dispersal distance (Figure 3b; Appendix Figures [Link], [Link], [Link], [Link], [Link]). On average, the population spread rate would increase by 3.2 centimeter per year if the maximal dispersal distance increases by one meter.

## 4 DISCUSSION

The study indicates that the population spread rate of the endangered species *M. smejkalii* is very limited and varies among different patches. The estimated maximal population spread rate was less than 6 cm/year, and the maximal seed dispersal distance was shorter than 2.5 m with very few seeds dispersing further than 0.5 m. Importantly, when the population growth rate was greater than one, the population spread rate depended more strongly on the maximal seed dispersal distance than on the population spread rate. Compared with the minimum distance of several meters that needs to be reached for *M. smejkalii* to colonize the closest suitable unoccupied sites, the population spread rate is critically low and the species suffers strong dispersal limitation. In the following, we discuss the significance of seed dispersal and local population dynamics to plant conservation, and recommend conservation strategies for *M. smejkalii*.

### 4.1 Long‐distance dispersal

The maximal seed dispersal distance strongly determines the population spread rate in *M*. *smejkalii*. This result is in line with previous studies that stress the critical significance of long‐distance dispersal (LDD) events to population spread (Jordano, 2017; Nathan, 2006; Nathan et al., 2002, 2008; Schurr et al., 2009; Trakhtenbrot et al., 2005). LDD events increase the population spread rate (Clark et al., 2001), habitat connectivity, and gene flow (Jordano, 2017). In contrast, insufficient LDD may threaten the survival of endemic species in fragmented landscapes (Trakhtenbrot et al., 2005). Due to critically limited seed dispersal ability, *M. smejkalii* may have difficulties to persist in its habitat, which became severally fragmented in the 1970s.

It is quite unlikely for *M. smejkalii* to colonize other potentially suitable sites without assisted colonization. *Minuartia smejkalii* seems to be autochorous, and dispersal distance could be mainly achieved by seeds falling from the plant (barochory) (Vittoz & Engler, 2007). The tiny seeds do not have any appendage that can facilitate wind dispersal or elaiosome that can attract animals that carry seeds away from plants. However, the species might be dispersed by epizoochory and endozoochory. Seeds dispersed by animals could reach further distances and may have higher germination rate and establishment probability (Wenny, 2001). What the other possible dispersal vectors could be and whether and how far other dispersal vectors can disperse seeds of *M. smejkalii* remain open questions.

While dispersal distance strongly affects population spread rate, postdispersal germination and survival determine what fraction of dispersal events will lead to “effective dispersal” (i.e., successful establishment of reproductive individuals) (Schupp et al., 2010). Despite a high germination rate (51% ± 30%) in the laboratory (Lozada‐Gobilard et al., 2020), *M*. *smejkalii* has low germination in the wild, and seeds can stay dormant for at least 10 years in the soil (Pánková unpublished data). Generally, seed dormancy can be an adaptive strategy of plants to avoid competition between offspring and mother plants (Baskin & Baskin, 2014), and this could be the case in *M*. *smejkalii* because an adult plant can live for at least 13 years and produce up to 64,000 seeds each year, and high germination would cause strong sib competition, as most of the seeds only land in the vicinity of the mother plant. Meanwhile, seed dormancy and germination could be affected by quality of maternal environment (Kildisheva et al., 2020; Thompson & Ooi, 2010; Tielbörger & Petrů, 2010). However, it remains to be tested whether seed dormancy and germination vary with distance to the mother plant in *M. smejkalii*.

### 4.2 Local population dynamics

The demographic analyses revealed that life‐history stage for which elasticity of population growth rate was greatest was patch‐specific. This is in line with previous studies showing that population growth rate often varies with environmental conditions (e.g., Dahlgren et al., 2016; Ehrlén et al., 2005; Jongejans et al., 2008). Accordingly, combination of the data across patches may cause that we will miss the most critical determinant of population growth rate in some patches, which could give rise to increased extinction risk of *M. smejkalii* at the meta‐population level. Our study thus clearly indicates that demographic data need to be collected for all the remnant patches for precise management of an endangered plant species.

Population growth rate also determines population persistence. All populations except Z2 are growing and can thus persist under current management regimes. In contrast, population Z2 may eventually go extinct without improvement of the management. However, the exact reasons for the low population growth rate in Z2 may be complex and are still largely unknown. Besides the low survival of adult plants, population growth rate could be negatively affected by the Allee effect (Keitt et al., 2001)—low‐density populations have reduced individual fitness, which in the long term could even lead to selection‐driven extinction (Gyllenberg et al., 2002). Future study could test whether increasing population density of Z2, for example, by adding seeds or plug seedlings, will increase population growth rate.

### 4.3 Population spread in fragmented habitats

Local population dynamics and dispersal are the main mechanistic drivers for spatial population spread (Hastings et al., 2005; Kot et al., 1996; Schreiber & Beckman, 2020; Skellam, 1951). As remnant habitats become smaller and more fragmented, considerable variation in local population dynamics and seed dispersal may occur among remnant patches, due to environmental heterogeneity and context dependency of local population dynamics and seed dispersal (Baden et al., 2021; Clobert et al., 2012). Accordingly, recent studies emphasize the significance of intraspecific variation in seed dispersal (Chen & Giladi, 2020; Snell et al., 2019) and local population dynamics (Yang et al., 2021) for predicting how plants respond to environmental change. Understanding this intraspecific variation is crucial in fragmented landscapes, because fragmentation could directly affect dispersal traits (Chen et al., 2020; Dener et al., 2021) and local population dynamics (Hobbs & Yates, 2003; Ibáñez et al., 2014; Opdam et al., 1993). Knowledge of local population dynamics and seed dispersal has been applied to inform management decisions to control the spread of invasive species (e.g., Jongejans et al., 2008; Shea et al., 2010), and to conserve rare species (Griffith & Forseth, 2005). However, in comparison with the sheer number of endangered plants, there is severe lack of data on context‐dependent local population dynamics and seed dispersal traits on endangered species. For example, complete demographic data are available for only a small and biased subset of species (Salguero‐Gómez et al., 2015), and context dependency has largely been ignored in currently available databases. Our results demonstrate that considerable variation in both local population dynamics and seed dispersal occurs among remnant patches of *M. smejkalii* and that this variation leads to different population growth and spread rates. Thus, our finding not only adds to growing understanding of mechanistic drivers of population spread in fragmented landscapes but also emphasizes the importance of long‐term data on local population dynamics and seed dispersal in remnant patches for plant conservation.

### 4.4 Conservation recommendations for *M. smejkalii*

In all remnant patches, efforts should be made to largely increase the dispersal ability of *M. smejkalii*. At the individual level, we recommend removing the surrounding vegetation to increase wind speed and thus dispersal distance. This could also increase population growth rate. We also recommend adding seeds or transplanting seedlings to Z2 to overcome the potential negative Allee effect. Furthermore, we recommend assisted colonization of potentially suitable sites to help the species to overcome the dispersal limitation. However, assisted colonization should be conducted with the utmost caution by predominantly using seeds or seedlings from the populations in the same region in order to avoid eroding the local genetic structure of extant populations (Stojanova et al., 2020). Particularly, we do not recommend using seeds from ex situ facilities with potential spontaneous hybridization, because the established hybrids could outcompete *M. smejkalii* in the wild (Lozada‐Gobilard et al., 2020).

### 4.5 CONCLUSION

The study indicates that knowledge of both seed dispersal and local population dynamics in remnant patches is important for understanding the potential of an endangered plant species to survive in the landscape and thus to predict its potential to colonize new suitable sites. Long‐term demographic data, and data on dispersal traits, dispersal vectors, and dispersal environments are essential to quantify local population dynamics, seed dispersal, and population spread. Population spread rate may strongly depend on maximal seed dispersal distance; hence, efforts made to increase seed dispersal ability could largely raise efficiency and effectiveness of conservation actions for critically endangered plant species.

## CONFLICT OF INTEREST

The authors have not declared any conflict of interest.

## AUTHOR CONTRIBUTIONS

**Jinlei Zhu:** Conceptualization (lead); Formal analysis (lead); Software (lead); Visualization (lead); Writing‐original draft (lead). **Karolína Hrušková:** Data curation (lead); Writing‐original draft (supporting). **Hana Pánková:** Conceptualization (supporting); Data curation (lead); Funding acquisition (lead); Investigation (lead); Supervision (supporting); Writing‐original draft (supporting). **Zuzana Munzbergova:** Conceptualization (supporting); Formal analysis (supporting); Funding acquisition (supporting); Methodology (supporting); Project administration (supporting); Supervision (lead); Writing‐original draft (supporting).

## Supporting information

Figure S1

Figure S2

Figure S3

Figure S4

Figure S5

Supplementary Material

## Data Availability

Data on seed dispersal and population spread are openly available from the Dryad Digital Repository (Zhu, 2022). https://doi.org/10.5061/dryad.tht76hf0f
