# Supplementary material for: Quantifying patch‐specific seed dispersal and local population dynamics to estimate population spread of an endangered plant species
Source: Ecol Evol. 2021 Sep 14;11(20):14070–8. doi: 10.1002/ece3.8116 (PMC8525078; doi:10.1002/ece3.8116)
Supplement: Supplementary file 6 — Supplementary Material [file ECE3-11-14070-s004.docx]

**Appendix**

**Table S1.** Number of measurements of seed release height and terminal velocity.

| Patch | Seed release height | | Terminal velocity | |
| --- | --- | --- | --- | --- |
|  | #individuals | #measurements | #individuals | #measurements |
| Z2 | 14 | 165 | 10 | 70 |
| Z4 | 10 | 150 | 5 | 45 |
| Z5 | 10 | 150 | 12 | 68 |
| Z6 | 13 | 130 | 6 | 33 |
| H | 23 | 165 | 9 | 58 |

**Table S2.** Input parameters of the WALD model. Values are given as means and standard deviations (in brackets).

| Patch | Terminal velocity, *v_t_* (m/s) | Release height, *H_r_* (cm) | Vegetation height, *h* (cm) | Horizontal wind velocity, *U_ref_* (m/s)* | | Reference height, *z_ref_* (m) |
| --- | --- | --- | --- | --- | --- | --- |
|  |  |  |  | Shape | Scale |  |
| Z2 | 1.47 (0.27) | 6.06 (2.49) | 3.0 (0.002) | 2.17 | 1.19 | 10 |
| Z4 | 1.61 (0.08) | 9.45 (2.88) | 3.0 (0.002) | 2.17 | 1.19 | 10 |
| Z5 | 1.65 (0.15) | 8.02 (2.62) | 3.0 (0.002) | 2.17 | 1.19 | 10 |
| Z6 | 1.56 (0.10) | 4.52 (1.88) | 3.0 (0.002) | 2.17 | 1.19 | 10 |
| H | 1.63 (0.14) | 5.79 (2.64) | 3.0 (0.002) | 3.80 | 2.84 | 10 |

*Weibull distributions are fitted to measurements.

**Table S3.** Dispersal distance (in m) at the patch level and across all patches.

| Patch | Minimum | 1% quantile | Median | Mean | 99% quantile | Maximum |
| --- | --- | --- | --- | --- | --- | --- |
| Z2 | 0 | 0.00015 | 0.00243 | 0.00437 | 0.02813 | 0.30086 |
| Z4 | 0 | 0.00017 | 0.00321 | 0.00579 | 0.03760 | 0.54229 |
| Z5 | 0 | 0.00017 | 0.00329 | 0.00593 | 0.03841 | 0.47897 |
| Z6 | 0 | 0.00020 | 0.00458 | 0.00843 | 0.05701 | 0.94296 |
| H | 0 | 0.00066 | 0.01071 | 0.02061 | 0.14503 | 2.43348 |
| Overall | 0 | 0.00027 | 0.00633 | 0.01268 | 0.09286 | 2.37115 |

**Table S4.** Probability of long-distance seed dispersal (LDD, threshold distance 0.5 m) at the patch level and across all patches.

| Patch | Minimum | 1% quantile | Median | Mean | 99% quantile | Maximum |
| --- | --- | --- | --- | --- | --- | --- |
| Z2 | 0 | 0 | 0 | 4.7e-10 | 0 | 0.002 |
| Z4 | 0 | 0 | 0 | 7.4e-8 | 0 | 0.188 |
| Z5 | 0 | 0 | 0 | 3.8e-8 | 0 | 0.072 |
| Z6 | 0 | 0 | 0 | 2.2e-6 | 0 | 0.261 |
| H | 0 | 0 | 0 | 1.5e-4 | 2e-5 | 0.992 |
| Overall | 0 | 0 | 0 | 3.2e-5 | 7.8e-9 | 0.973 |

**Table S5.** Coefficient of determination (*R*^2^) of linear regression models between the rate of population spread (wave speed) and population growth rate (λ) and the maximal dispersal distance in each patch. In the models, explanatory variables (λ and the maximal dispersal distance) were scaled.

| Patch | Partial *R*^2^ | |
| --- | --- | --- |
|  | Population growth rate | Maximal dispersal distance |
| Z2 | 0.016 | 0.881 |
| Z4 | 0.161 | 0.554 |
| Z5 | 0.213 | 0.377 |
| Z6 | 0.027 | 0.886 |
| H | 0.051 | 0.830 |


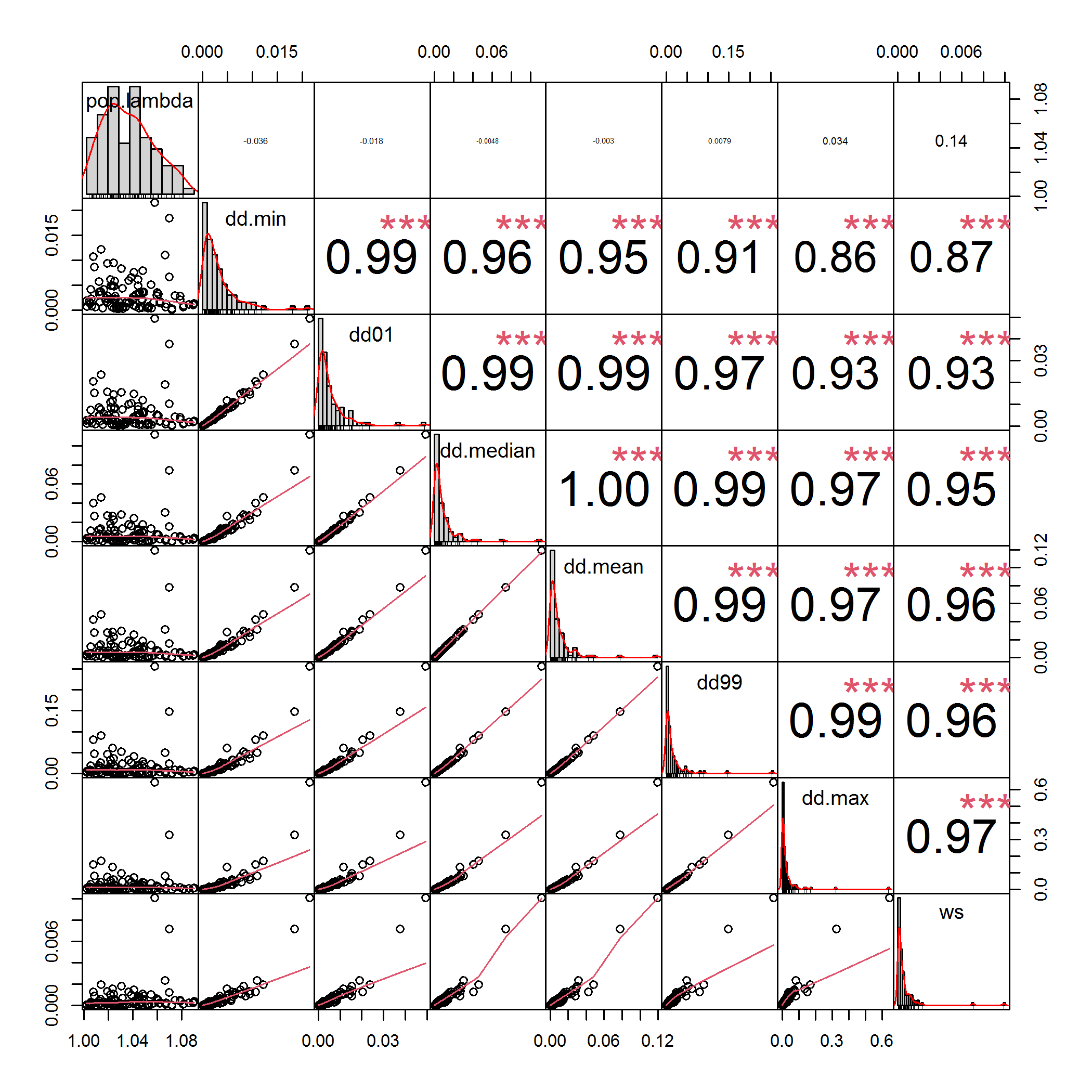


**Fig. S1.** Correlation matrix between wave speed and population growth rate as well as various statistics of dispersal distance for the patch Z2. pop.lambda: The population growth rate (λ); dd.min: Minimal dispersal distance; dd01: 1% quantile of dispersal distance; dd.median: Median dispersal distance; dd.mean: Mean dispersal distance; dd99: 99% quantile of dispersal distance; dd.max: Maximal dispersal distance; ws: Wave speed. ***P<0.001.


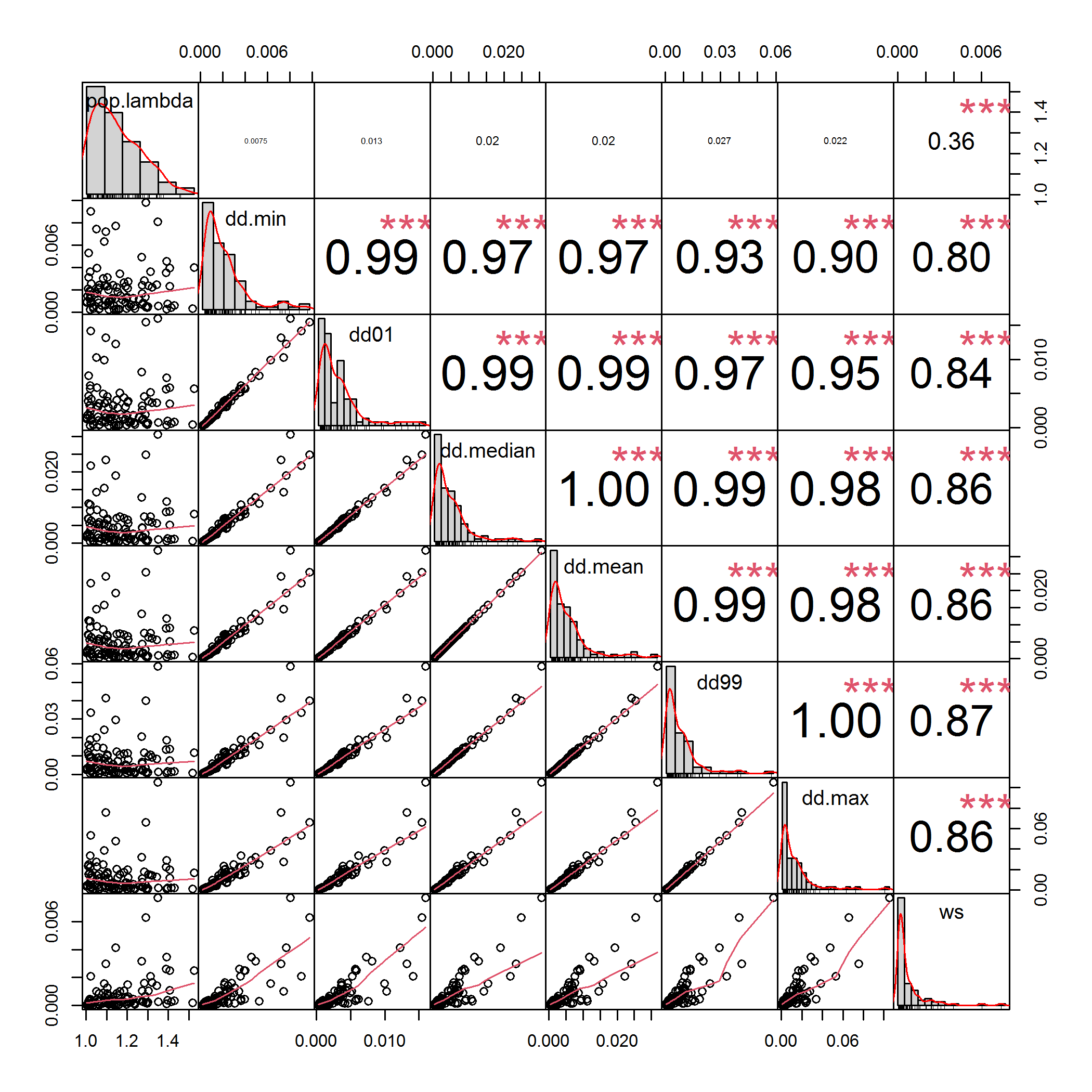


**Fig. S2.** Correlation matrix between wave speed and population growth rate as well as various statistics of dispersal distance for the patch Z4. pop.lambda: The population growth rate (λ); dd.min: Minimal dispersal distance; dd01: 1% quantile of dispersal distance; dd.median: Median dispersal distance; dd.mean: Mean dispersal distance; dd99: 99% quantile of dispersal distance; dd.max: Maximal dispersal distance; ws: Wave speed. ***P<0.001.


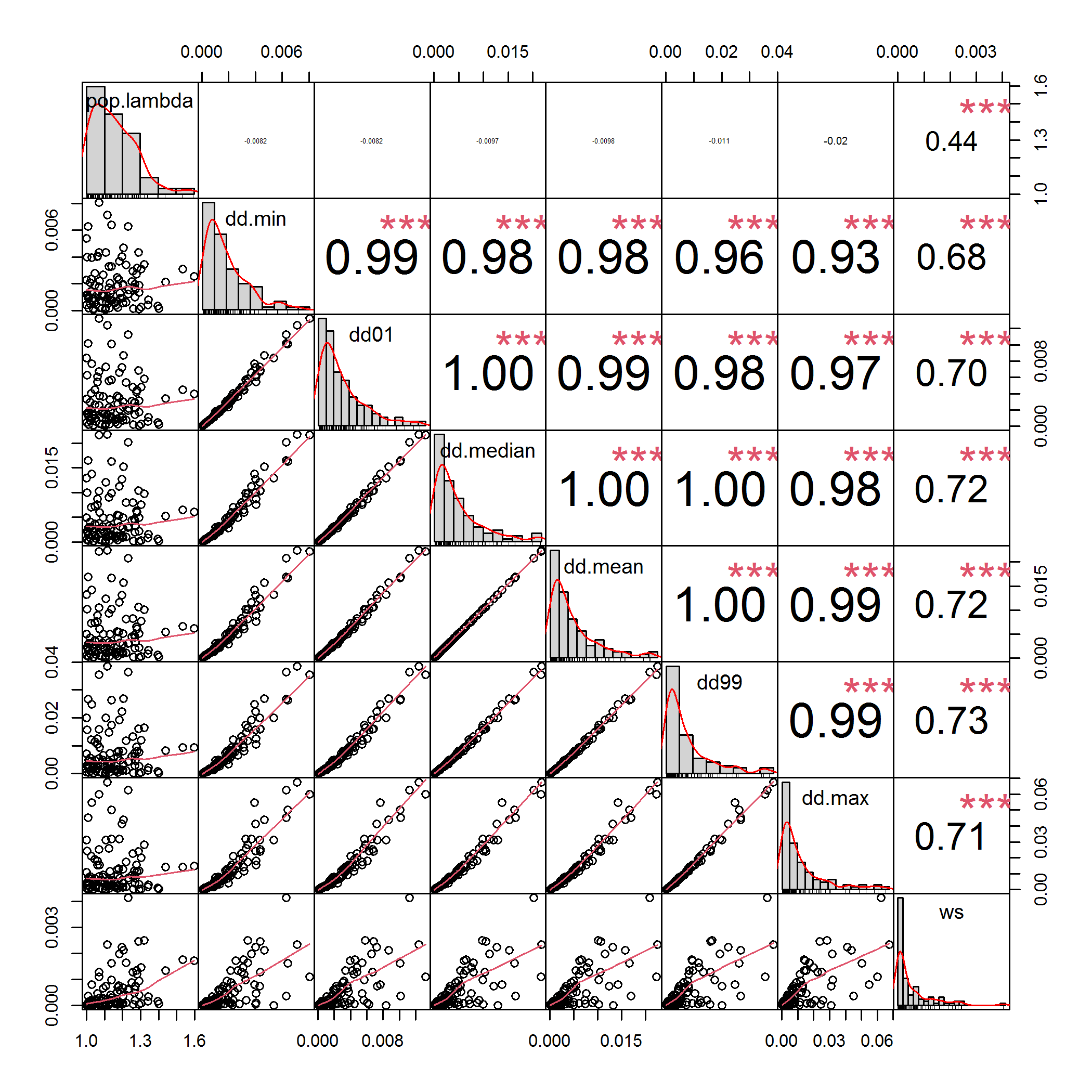


**Fig. S3.** Correlation matrix between wave speed and population growth rate as well as various statistics of dispersal distance for the patch Z5. pop.lambda: The population growth rate (λ); dd.min: Minimal dispersal distance; dd01: 1% quantile of dispersal distance; dd.median: Median dispersal distance; dd.mean: Mean dispersal distance; dd99: 99% quantile of dispersal distance; dd.max: Maximal dispersal distance; ws: Wave speed. ***P<0.001.


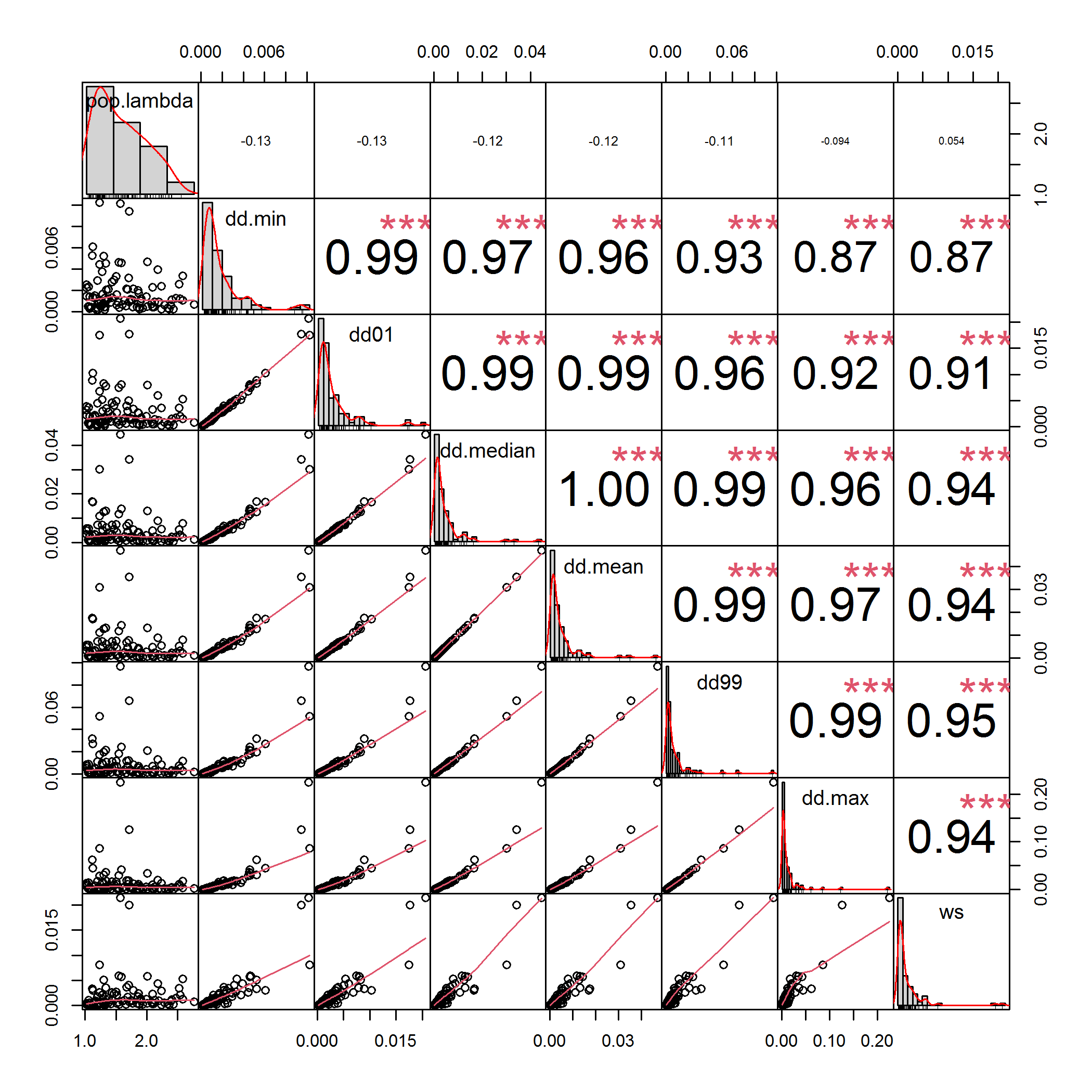


**Fig. S4.** Correlation matrix between wave speed and population growth rate as well as various statistics of dispersal distance for the patch Z6. pop.lambda: The population growth rate (λ); dd.min: Minimal dispersal distance; dd01: 1% quantile of dispersal distance; dd.median: Median dispersal distance; dd.mean: Mean dispersal distance; dd99: 99% quantile of dispersal distance; dd.max: Maximal dispersal distance; ws: Wave speed. ***P<0.001.


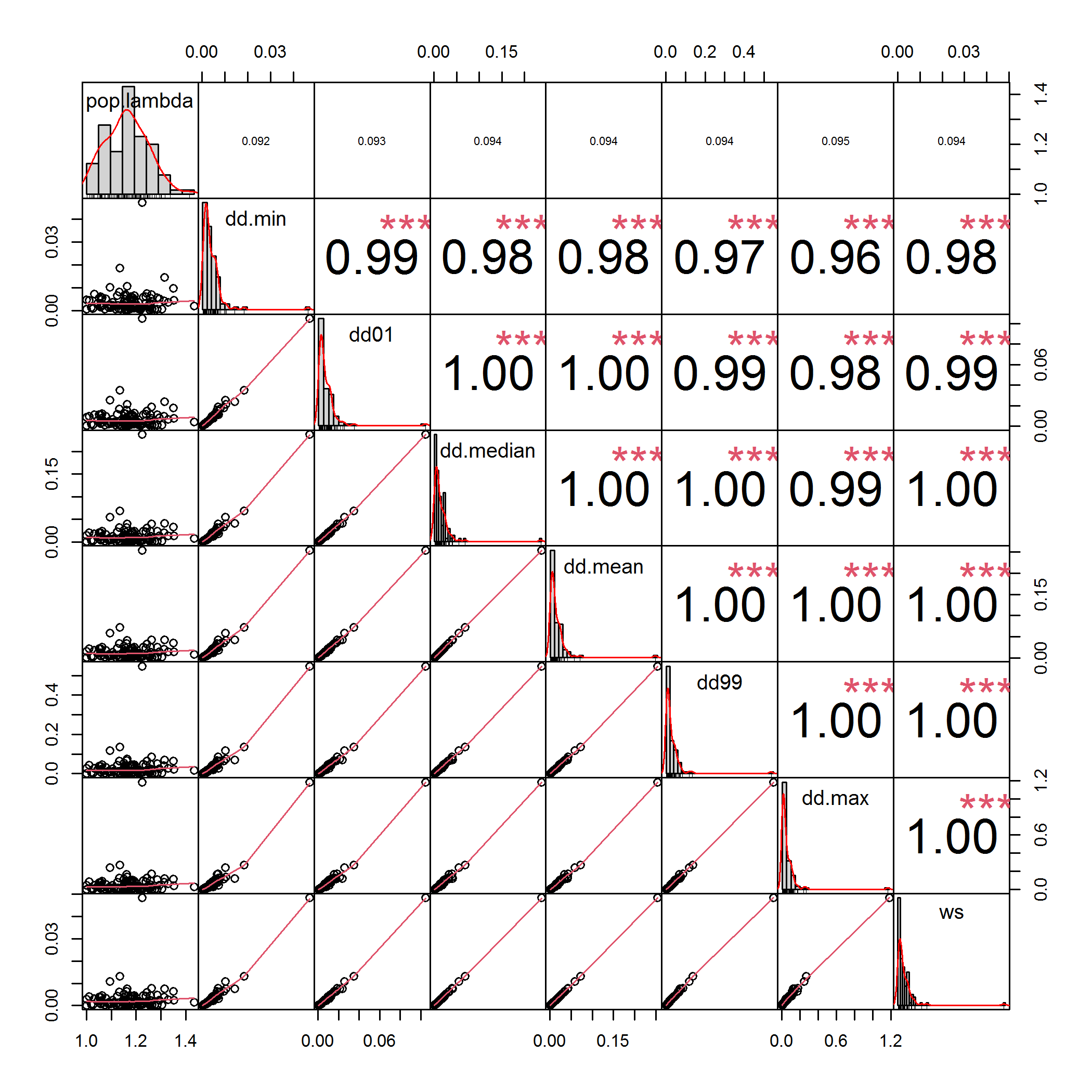


**Fig. S5.** Correlation matrix between wave speed and population growth rate as well as various statistics of dispersal distance for the patch H. pop.lambda: The population growth rate (λ); dd.min: Minimal dispersal distance; dd01: 1% quantile of dispersal distance; dd.median: Median dispersal distance; dd.mean: Mean dispersal distance; dd99: 99% quantile of dispersal distance; dd.max: Maximal dispersal distance; ws: Wave speed. ***P<0.001.

**Appendix S1: Population projection matrices**

Stage-structured population projection matrices for *M. smejkalii* in each patch in 2016, 2017, and 2018. The transitions involving seed dispersal, that is, juvenile to seedling and adult to seedling, are given in bold.

Patch Z2 in 2016:

|  | seedling | juvenile | adult |
| --- | --- | --- | --- |
| seedling | **0.005598824** | **0.042865791** | **0.355164886** |
| juvenile | 0.328125 | 0.5 | 0.097222222 |
| adult | 0.078125 | 0.294871795 | 0.763888889 |

Patch Z2 in 2017:

|  | seedling | juvenile | adult |
| --- | --- | --- | --- |
| seedling | **0.03624894** | **0.091464026** | **0.455738676** |
| juvenile | 0.454545455 | 0.561797753 | 0.11627907 |
| adult | 0.03030303 | 0.146067416 | 0.779069767 |

Patch Z2 in 2018:

|  | seedling | juvenile | adult |
| --- | --- | --- | --- |
| seedling | **0.000200969** | **0.067326477** | **0.330008877** |
| juvenile | 0.484848485 | 0.451612903 | 0.277777778 |
| adult | 0.090909091 | 0.064516129 | 0.5 |

Patch Z4 in 2016:

|  | seedling | juvenile | adult |
| --- | --- | --- | --- |
| seedling | **0** | **0.032541322** | **0.260920897** |
| juvenile | 0.225 | 0.291666667 | 0 |
| adult | 0.020408163 | 0.25 | 0.904761905 |

Patch Z4 in 2017:

|  | seedling | juvenile | adult |
| --- | --- | --- | --- |
| seedling | **0.013610831** | **0.028564268** | **0.111111494** |
| juvenile | 0.666666667 | 0.75 | 0.130434783 |
| adult | 0 | 0.05 | 0.826086957 |

Patch Z4 in 2018:

|  | seedling | juvenile | adult |
| --- | --- | --- | --- |
| seedling | **0.045989173** | **1.001479735** | **2.539174457** |
| juvenile | 0.5 | 0.882352941 | 0.222222222 |
| adult | 0 | 0.058823529 | 0.611111111 |

Patch Z5 in 2016:

|  | seedling | juvenile | adult |
| --- | --- | --- | --- |
| seedling | **0.039034969** | **0.163865546** | **1.895798319** |
| juvenile | 0.548387097 | 0.571428571 | 0.5 |
| adult | 0.064516129 | 0.428571429 | 0.5 |

Patch Z5 in 2017:

|  | seedling | juvenile | adult |
| --- | --- | --- | --- |
| seedling | **0.017336847** | **0.067038458** | **0.169014715** |
| juvenile | 0.75 | 0.695652174 | 0.142857143 |
| adult | 0 | 0.086956522 | 0.857142857 |

Patch Z5 in 2018:

|  | seedling | juvenile | adult |
| --- | --- | --- | --- |
| seedling | **0.014359491** | **0.019266461** | **0.051614383** |
| juvenile | 0 | 0.476190476 | 0.25 |
| adult | 0 | 0.238095238 | 0.625 |

Patch Z6 in 2016:

|  | seedling | juvenile | adult |
| --- | --- | --- | --- |
| seedling | **0.225297** | **0.259161425** | **2.165351** |
| juvenile | 0.136364 | 0.375 | 0.136364 |
| adult | 0.272727 | 0.25 | 0.772727 |

Patch Z6 in 2017:

|  | seedling | juvenile | adult |
| --- | --- | --- | --- |
| seedling | **0.0580176** | **0.026594042** | **0.114737** |
| juvenile | 0.4035088 | 0.636363636 | 0.115385 |
| adult | 0.1403509 | 0.272727273 | 0.769231 |

Patch Z6 in 2018:

|  | seedling | juvenile | adult |
| --- | --- | --- | --- |
| seedling | **0.859595615** | **1.788487677** | **10.08229596** |
| juvenile | 0.542168675 | 0.424242424 | 0.214285714 |
| adult | 0.096385542 | 0.181818182 | 0.464285714 |

Patch H in 2016:

|  | seedling | juvenile | adult |
| --- | --- | --- | --- |
| seedling | **0.047123658** | **0.026071638** | **0.815788804** |
| juvenile | 0.275423729 | 0.5625 | 0.109589041 |
| adult | 0.313559322 | 0.3125 | 0.767123288 |

Patch H in 2017:

|  | seedling | juvenile | adult |
| --- | --- | --- | --- |
| seedling | **0.060060445** | **0.034509995** | **0.487725656** |
| juvenile | 0.452054795 | 0.467889908 | 0.100671141 |
| adult | 0.356164384 | 0.422018349 | 0.859060403 |

Patch H in 2018:

|  | seedling | juvenile | adult |
| --- | --- | --- | --- |
| seedling | **0.104493248** | **0.094959853** | **0.628548307** |
| juvenile | 0.5 | 0.75257732 | 0.255 |
| adult | 0.290322581 | 0.12371134 | 0.665 |

**Appendix S2: Simulation of dispersal distance with the WALD mechanistic model**

The WALD model is an analytical mechanistic model derived from a simplified 3-D stochastic dispersion model that retains the essential physics contained within the more computationally intensive coupled Eulerian-Lagrangian closure (CELC) model (Nathan et al. 2002). The analytical model reduces to the following WALD (or inverse Gaussian) distribution (eqn 5b in Katul et al. 2005) that describes the probability density of dispersal distances x

$p\left( x \right)=\left( \frac{\lambda}{2\pi x^{3}} \right)^{1/2}exp\left[ -\frac{\lambda\left( x-\mu\right)^{2}}{2\mu^{2}x} \right]$ (1)

where $\mu$ and $\lambda$ are dispersal kernel parameters, and $\mu={H_{r}U}/{V_{t}}$, $\lambda=\left( {H_{r}}/\sigma\right)^{2}$, *H_r_* is seed release height, *U* is the horizontal wind speed, *V_t_* is seed terminal velocity, and σ is a turbulent flow parameter reflecting wind speed variation (Katul et al. 2005, Skarpaas and Shea 2007).

$\sigma^{2}= \frac{4{\sigma_{w}}^{4}}{C_{0}\varepsilon U}$ (2)

where $\sigma_{w}$ is the vertical turbulence, $C_{0}$ is Kolmogorov constant (3.125), and $\varepsilon$ is the dissipation rate. Estimating $\varepsilon$ is challenging within the canopy (Katul et al. 2005), but we are mainly concerned with turbulent flow above the canopy, so we can assume $\varepsilon= {{u_{*}}^{3}}/{K\left( z-d \right)}$ and the constant $A_{w}\equiv{\sigma_{w}}/{u_{*}}\approx1.3$, where *z* is height above ground, *d* is zero-plane displacement distance (Skarpaas and Shea 2007). From these assumptions and equation (2), we obtain

$\sigma=2{A_{w}}^{2}\sqrt{\frac{K\left( z-d \right)u_{*}}{C_{0}U}}$ (3)

In the WALD simulation, each seed released was dispersed by randomly drawing a distance from the probability distribution function generated by the WALD model with parameters for wind speed, *H_r_* and *V_t_*. Wind speed was drawn from a Weibull distribution determined by wind measurements at two weather stations close to in Želivka and Hrnčíře at the reference height of 10 m.

**References**

Katul, G. G., et al. 2005. Mechanistic analytical models for long-distance seed dispersal by wind. - Am Nat 166: 368-81.

Nathan, R., et al. 2002. Mechanisms of long-distance dispersal of seeds by wind. - Nature 418: 409-13.

Skarpaas, O. and Shea, K. 2007. Dispersal patterns, dispersal mechanisms, and invasion wave speeds for invasive thistles. - Am Nat 170: 421-30.

**Appendix S3: Estimation of rate of population spread with matrix population model**

To estimate the population spread rate we followed Bullock et al. (2012) and Hemrová et al. (2017), using the analytical wavespeed model of Neubert and Caswell (2000), an integrodifference equation combining a demographic matrix with a dispersal kernel (Gilbert et al. 2014). The model simulates a population spreading in one dimension from a starting point in discrete times. According to Neubert and Caswell (2000), population density at location *x* at time *t+1* is

$$n\left( x,t+1 \right)=\int_{-\infty}^{\infty} \left[ K\left( x-y \right)\circ B_{n} \right]n\left( y,t \right)dy$$

where $\circ$ is the Hadamard product operator, *B_n_* is a stage-structured population projection matrix describing density-dependent population growth at location *y*, *K(x – y)* is a matrix of dispersal kernels describing the set of probabilities of the relocation from *y* to *x* of individuals undergoing each demographic transition, with the assumption that dispersal from *y* to *x* depends only on the relative locations of the two points.

Calculation of the wavespeed requires a projection matrix representing demography at low density (i.e. at the fore front of the spreading population; ***A*** = ***B****_0_*). A matrix ***M****(s)* describes the dispersal kernel for each demographic transition in terms of a moment generating function (MGF). The WALD model has an analytical MGF and thus dispersing elements of the matrix *M(s)* are as follows

$$m_{ij}\left( s \right)=exp\left[ \frac{\lambda^{'}\left( u \right)}{\mu^{'}\left( u \right)}\left( 1-\sqrt{1-\frac{2\mu^{'}\left( u \right)^{2}\left( s \right)}{\lambda^{'}\left( u \right)}} \right) \right]$$

where *s* describes the shape of the population wave (Neubert and Caswell 2000); for non-dispersing transitions *m_ij_(s)* = 1. Under this model a population forms a wave of a constant shape that advances at constant speed *c** (the wavespeed), which can be derived analytically by

$$c^{*}=\min_{s>0} \left( \frac{1}{s}\ln\rho\left( s \right) \right)$$

where *ρ* is the dominant eigenvalue of $\boldsymbol{A}\circ\boldsymbol{M}(s)$.

**References**

Bullock JM, White SM, Prudhomme C, Tansey C, Perea R, Hooftman, DAP. 2012. Modelling spread of British wind-dispersed plants under future wind speeds in a changing climate. Journal of Ecology **100**: 104-115.

Gilbert MA, White SM, Bullock JM, Gaffney EA. 2014. Spreading speeds for stage structured plant populations in fragmented landscapes. Journal of Theoretical Biology **349**:135-149.

Hemrová L, Bullock JM, Hooftman DAP, White SM, Münzbergová Z. 2017. Drivers of plant species’ potential to spread: the importance of demography versus seed dispersal. Oikos **126**:1493-1500.

Neubert MG, Caswell H. 2000. Demography and dispersal: calculation and sensitivity analysis of invasion speed for structured populations. Ecology **81**:1613-1628.

**Appendix S4: Elasticity matrices of λ across all patches and at the patch level**

Elasticity matrix across all patches was calculated with a mean population projection matrix over three years (2016-2018) and across all the five patches. Elasticity matrix for each patch was calculated with a mean projection matrix over three years (2016-2018) for that patch.

**Elasticity matrix of λ across all patches**

|  | seedling | juvenile | adult |
| --- | --- | --- | --- |
| seedling | 0.016743 | 0.052822 | 0.173659 |
| juvenile | 0.148132 | 0.133487 | 0.027801 |
| adult | 0.07835 | 0.12311 | 0.245895 |

**Elasticity matrix of λ for each patch**

Patch Z2:

|  | seedling | juvenile | adult |
| --- | --- | --- | --- |
| seedling | 0.001562 | 0.014614 | 0.082386 |
| juvenile | 0.073794 | 0.171857 | 0.055589 |
| adult | 0.023205 | 0.114769 | 0.462224 |

Patch Z4:

|  | seedling | juvenile | adult |
| --- | --- | --- | --- |
| seedling | 0.00292 | 0.067389 | 0.084505 |
| juvenile | 0.147407 | 0.263843 | 0.022135 |
| adult | 0.004488 | 0.102152 | 0.305162 |

Patch Z5:

|  | seedling | juvenile | adult |
| --- | --- | --- | --- |
| seedling | 0.002472 | 0.015328 | 0.090892 |
| juvenile | 0.098127 | 0.230953 | 0.082914 |
| adult | 0.008093 | 0.165713 | 0.305508 |

Patch Z6:

|  | seedling | juvenile | adult |
| --- | --- | --- | --- |
| seedling | 0.088145 | 0.054305 | 0.240842 |
| juvenile | 0.1222 | 0.055038 | 0.013295 |
| adult | 0.172946 | 0.08119 | 0.172039 |

Patch H:

|  | seedling | juvenile | adult |
| --- | --- | --- | --- |
| seedling | 0.010959 | 0.009265 | 0.161029 |
| juvenile | 0.069887 | 0.116791 | 0.042644 |
| adult | 0.100407 | 0.103266 | 0.385753 |
